# Growth Performance and Carcass Traits in Purebred Apulo-Calabrese and Crossbred (Apulo-Calabrese × Goland) Pigs Fed Linseed and Grape Pomace Powder Naturally Rich in Antioxidants

**DOI:** 10.3390/vetsci13070703

**Published:** 2026-07-18

**Authors:** Anna Antonella Spina, Maria Elena Furfaro, Vincenzo Chiofalo, Alessandro Madeo, Domenico Britti, Cinzia Lucia Randazzo, Georgiana Bosco, Valeria Maria Morittu

**Affiliations:** 1 Department of Medical and Surgical Sciences, University “Magna Graecia” of Catanzaro, 88100 Catanzaro, Italy; aa.spina@unicz.it; 2 Meat and Agribusiness Chain Research Consortium, 98168 Messina, Italy; mariaelena.furfaro@gmail.com; 3 Department of Veterinary Sciences, University of Messina, 98168 Messina, Italy; vincenzo.chiofalo@unime.it; 4 Agrimad srl, 87069 San Demetrio Corone, Italy; alessandro.madeo@filieramadeo.it; 5 Department of Experimental and Clinical Medicine, University “Magna Graecia” of Catanzaro, 88100 Catanzaro, Italy; britti@unicz.it; 6 Department of Agriculture, Food and Environment, University of Catania, 95123 Catania, Italy; cinzia.randazzo@unict.it (C.L.R.); georgiana.bosco@phd.unict.it (G.B.)

**Keywords:** Apulo-Calabrese pigs, local breeds, growth performance, carcass traits, linseed, grape pomace, natural feed additives

## Abstract

Preserving the Apulo-Calabrese (AC), an endangered Italian autochthonous pig breed, requires sustainable nutritional strategies that improve pork quality without compromising productive performance. This study evaluated the effects of dietary supplementation with extruded linseed, alone or combined with grape pomace powder, rich in antioxidants, on growth performance and carcass traits in purebred Apulo-Calabrese (AC) pigs and Apulo-Calabrese × Goland (AC × G) crossbred pigs. Two independent feeding trials were conducted using three dietary treatments: a control diet (CTR), a diet containing 5% extruded linseed (SL), and a diet containing 5% extruded linseed plus 0.2% grape pomace powder rich in polyphenols (SLXI). Thirty-six purebred AC pigs reared outdoors, and 30 AC × G crossbred pigs reared indoors were included in the study. Body weight (BW) and average daily gain (ADG) were recorded throughout the trials, while hot carcass weight (HCW), carcass yield (CY), pH45, pH24, and backfat thickness (BF) were evaluated at slaughter. Data were analyzed separately for the two trials using Linear Mixed Models. Feed intake was complete in all groups, indicating good diet palatability. In purebred AC pigs, dietary treatment significantly affected overall ADG, with CTR pigs showing higher ADG than SLXI animals, while SL displayed intermediate values not significantly different from either CTR or SLXI. BW and carcass traits were not significantly influenced by diet. In addition, a significant Diet × Time interaction was detected for ADG (*p* = 0.010), indicating diet-dependent growth patterns over time. Sex did not significantly affect BW, ADG, or carcass traits in purebred pigs. In crossbred AC × G pigs, dietary treatments could not be evaluated inferentially due to the lack of pen-level replication; therefore, growth and carcass data are presented descriptively. However, Sex significantly affected carcass yield, with males showing higher CY than females (*p* = 0.004), while BW, ADG, and the remaining carcass traits were not influenced by Sex. The results suggest that extruded linseed, alone or combined with low-dose grape pomace powder, can be included in diets for purebred and crossbred pigs without compromising growth performance or basic carcass traits. These findings support the use of powdered grape pomacein feeding strategies aimed at the valorization and conservation of Apulo-Calabrese pig genetic resources. Further studies are needed to evaluate the effects of these dietary strategies on meat fatty acid composition, oxidative stability, sensory quality, and shelf life.

## Simple Summary

Pig farming is increasingly expected to support the conservation of local breeds while maintaining acceptable productive performance. The Apulo-Calabrese pig is an Italian native breed with important cultural and agricultural value, but more information is needed to support its conservation and productive use. In this study, purebred Apulo-Calabrese pigs and crossbred pigs were fed diets enriched with linseed, as a source of polyunsaturated fatty acids, and grape pomace powder naturally rich in antioxidants. The results showed that these dietary supplements did not negatively affect feed intake, body weight, growth, or most carcass characteristics in purebred Apulo-Calabrese subjects. In crossbred pigs, although dietary effects could not be tested statistically and results were therefore interpreted only descriptively, numerical data showed some improvements in hot carcass weight and carcass yield compared with the group receiving the standard diet. Importantly, the grape-derived supplement did not produce negative effects often associated with plant tannins. These findings suggest that the natural plant-based feed ingredients evaluated in this study can be used safely in pig diets and preserve valuable local breeds such as the Apulo-Calabrese pig.

## 1. Introduction

Pig production currently faces the challenge of combining sustainability, conservation of animal biodiversity, and the production of healthier and higher-quality meat products [1]. Pork remains one of the most consumed meats worldwide, and consumer demand is increasingly oriented toward products characterized not only by good sensory and technological properties, but also by nutritional value, environmental sustainability, animal welfare, and strong links to local traditions and geographical origin [2,3].

In this context, local pig breeds represent valuable genetic resources because of their adaptation to marginal environments, disease resistance, and contribution to traditional food chains and rural economies [4]. The Apulo-Calabrese pig is an Italian autochthonous breed characterized by adaptability to extensive production systems, rusticity, and the production of high-quality traditional pork products. Although its growth performance is generally lower than that of highly selected commercial genotypes, the breed represents an important genetic resource for biodiversity conservation [5].

The Apulo-Calabrese pig is considered a genetic resource at risk of extinction, and its conservation requires sustainable production strategies capable of improving its economic competitiveness without compromising its identity and productive characteristics [6,7]. Moreover, local breeds are increasingly recognized as important tools for improving livestock system resilience under climate change scenarios due to their adaptability to low-input farming systems and harsh environmental conditions [8,9].

Growing consumer awareness of the relationship between diet and health has also stimulated interest in functional animal-derived foods, including pork enriched with beneficial fatty acids and natural antioxidant compounds [10]. Feeding strategies are recognized as one of the main factors influencing pork quality, particularly fatty acid composition, oxidative stability, and technological traits [11,12]. However, increased concentrations of polyunsaturated fatty acids may also enhance lipid oxidation processes, negatively affecting shelf life and sensory quality [13]. To overcome these limitations, agro-industrial by-products rich in antioxidants have gained increasing attention in animal nutrition. Among these, grape pomace is a sustainable source of polyphenols with antioxidant, antimicrobial, and anti-inflammatory properties, and it also contributes to circular economy approaches through the valorization of winery residues [14,15]. Several studies have demonstrated the potential of grape-derived polyphenols to improve oxidative stability and quality traits in animal products [16,17]. However, controversial effects on growth performance and nutrient digestibility have also been reported, mainly due to the presence of tannins, which may exert negative nutritional effects when included in the diet [18,19]. Consequently, identifying appropriate supplementation strategies capable of improving pork quality without compromising productive performance remains an important research objective.

Therefore, the present study aimed to evaluate the effects of dietary supplementation with extruded linseed and grape pomace powder on growth performance and carcass traits in purebred Apulo-Calabrese pigs and Apulo-Calabrese × Goland crossbred pigs. Apulo-Calabrese × Goland crossbred pigs are intended to combine the adaptability and product quality traits of the local breed with the greater growth potential of commercial genetic lines. In particular, the study investigated whether the combined use of an essential fatty acid source and grape pomace powder could constitute a sustainable nutritional strategy capable of improving productive performance and carcass quality without inducing negative effects associated with polyphenol-rich feed additives. Furthermore, the study sought to assess the applicability of these feeding strategies in both conservation-oriented production systems involving an endangered local breed and in crossbreeding systems aimed at maintaining productive efficiency.

## 2. Materials and Methods

### 2.1. Ethical Statement

All animal procedures were conducted in accordance with European legislation on the protection of animals used for scientific purposes [20]. The study was carried out following standard farming and veterinary management; therefore, ethical approval for the use of experimental animals was not required.

### 2.2. Experimental Design

The study consisted of two independent feeding trials conducted to evaluate the effects of dietary supplementation with extruded linseed and grape pomace powder on growth performance and carcass traits in purebred Apulo-Calabrese (AC) pigs and Apulo-Calabrese × Goland (AC × G) crossbred pigs. In both trials, animals were randomly assigned to one of three dietary treatments: a control diet without supplementation (CTR), a diet supplemented with 5% extruded linseed (SL), and a diet supplemented with 5% extruded linseed plus 0.2% powered grape pomace rich in polyphenols (SLXI).

Experimental diets were administered during the growing and finishing phases, and productive performance and slaughter traits were evaluated throughout the experimental period. In both trials, slaughter time was determined according to the commercial requirements of the hosting farm, which processes hams for dry-cured production and therefore adopts a target live weight of approximately 135 kg to ensure adequate hind-limb muscular development.

### 2.3. Animals and Housing

The two independent feeding trials were performed at a commercial farm located in the San Demetrio Corone municipality (Calabria, Southern Italy).

The farm operated under standard commercial management conditions. In both trials, pigs had ad libitum access to water through nipple drinkers (approximately one drinker per 10 pigs) and were fed using stainless-steel wet feeders, providing at least 0.60 m of feeder space per pig. Pens were equipped with chopped straw bedding and featured a combination of solid and partially slatted concrete flooring. Animals had access to both a sheltered resting area and an open exercise space, while buildings relied on natural ventilation. During the experimental period, indoor temperatures ranged approximately between 18 and 25 °C, whereas outdoor ambient temperatures varied seasonally from approximately 10 °C in February to 25–30 °C in May–June.

#### 2.3.1. Purebred Apulo-Calabrese Trial

The purebred trial was carried out on 36 Apulo-Calabrese female (F) and neutered males (M) pigs reared outdoors. At the beginning of the trial, animals were 203 ± 21 d of age and had an initial body weight of 63 ± 10 kg.

Animals were randomly assigned to three experimental groups that were homogeneous with respect to body weight, age, and sex: CTR (*n* = 12): control diet; SL (*n* = 12): diet supplemented with 5% extruded linseed; SLXI (*n* = 12): diet supplemented with 5% extruded linseed and 0.2% grape pomace powder. Pigs were housed in six outdoor paddocks of equal size, two paddocks per group with six animals per paddock, including both sexes. Pigs were housed in six outdoor paddocks of equal size, two paddocks per dietary treatment, with six pigs per paddock, including both sexes. Each paddock measured approximately 50 m^2^, providing about 2.5 m^2^ of available space per pig. Each paddock included a covered resting area and an outdoor exercise area.

The feeding trial, after 13 days of adaptation diet, lasted 140 days, and animals were sacrificed at 356 ± 21 d of age.

#### 2.3.2. Crossbred Apulo-Calabrese × Goland Trial

The crossbred trial involved 30 Apulo-Calabrese × Goland females (F) and neutered males (M) reared indoors, having 121 ± 3 d of age and 58 ± 8 kg of body weight.

Animals were randomly allocated to three experimental groups, which were homogeneous in body weight, age, and sex: CTR (*n* = 10): control diet; SL (*n* = 10): diet supplemented with 5% extruded linseed; SLXI (*n* = 10): diet supplemented with 5% extruded linseed and 0.2% grape pomace powder.

Each group was housed in a separate indoor pen within the same building. Pens measured approximately 24 m^2^, providing approximately 1.5 m^2^ of floor space per pig.

Before the beginning of the experimental trial, pigs underwent a 13-day adaptation period, and the feeding trial lasted 97 days. Animals were slaughtered at 231 ± 3 d of age.

### 2.4. Experimental Diets

Experimental diets were formulated according to the nutritional requirements for growing–finishing pigs established by the National Research Council (NRC) [21]. In both trials, two feeding phases were adopted according to body weight: growing (50–100 kg body weight) and finishing (100–140 kg body weight).

Three experimental diets were tested: a conventional control diet without supplementation (CTR), a diet supplemented with 5% extruded linseed (SL), and a diet supplemented with 5% extruded linseed plus 0.2% grape pomace powder rich in polyphenols (12,200 mg gallic acid equivalents/100 g) (SLXI).

In both feeding trials, pigs underwent a 13-day adaptation period before the beginning of the experimental phase, during which all animals received the control diet.

During the experimental period, diets were administered twice daily in mash form mixed with water at a feed-to-water ratio of 1:3. Feed allowance was adjusted according to the production phase and genetic group.

In the purebred Apulo-Calabrese trial, pigs received 2400 g/head/day during the first phase (T0–T37), 2700 g/head/day during the second phase (T37–T71), 3000 g/head/day during the third phase (T71–T116), and 3200 g/head/day during the final finishing phase (T116–T140).

In the crossbred Apulo-Calabrese × Goland trial, pigs received 2260 g/head/day during the first experimental phase (T0–T18), 2400 g/head/day from T18 to T56, and 2800 g/head/day during the finishing phase (T56–T97). Fresh and clean water was provided ad libitum throughout the study.

The chemical characteristics of the diets used in the study were determined according to the AOAC methods [22], while total polyphenol content (TPC) was extracted and quantified according to the Folin–Ciocalteu method, as previously described by Maurotti et al. [23]. Antioxidant capacity was assessed using the DPPH radical scavenging assay, following the procedure reported by Mare et al. [24]. Total lipids were extracted according to the method of Bligh and Dyer [25], while fatty acid methyl esters (FAMEs) were prepared following the procedure described by Christie [26]. FAMEs were subsequently analyzed by gas chromatography (Shimadzu GC-2010 Pro, Shimadzu Corporation, Kyoto, Japan).

The analysis of each chemical parameter was replicated twice for each by-product, and the results were reported as the mean of the two replicates.

As the experimental feeds were commercial formulations, the exact inclusion rates of individual ingredients were not disclosed by the manufacturer. Therefore, the ingredient composition is reported qualitatively in decreasing order of inclusion, while the analyzed chemical composition of each diet is provided in Table 1 and Table 2. Two diet formulations were used according to the production phase: growing diet, from 50 to 100 kg body weight, and finishing diet, from 100 to 140 kg body weight. The control diets were mainly based on corn, barley, soybean meal, wheat bran, citrus pulp, molasses, and mineral sources. In the SL diets, extruded linseed was included at 5%, together with cereal grains, legumes, beet pulp, molasses, and mineral sources. The SLXI diets had the same formulation as the SL diets, with the addition of 0.2% grape pomace powder, consisting of dried and powdered grape pomace, as a natural source of polyphenols.

### 2.5. Growth Performance

Initial body weight (BW) was recorded individually at day 0 (T0). Throughout the study period, individual BW was monitored at approximately monthly intervals for purebred AC pigs (T37, T71, T116, and T140 d) and biweekly for AC × G pigs (T18, T35, T56, T72, and T97 d).

Average daily gain (ADG) was calculated for each experimental phase and for the overall trial period. Feed intake was monitored throughout the study, and the feed conversion ratio (FCR) was calculated as the ratio between average feed intake and ADG.

### 2.6. Slaughter and Carcass Traits

At the end of each feeding trial, all pigs were slaughtered in a commercial slaughterhouse following standard industrial procedures and current European regulations on animal welfare during transport and slaughter. Animals were fasted for 12 h before slaughter. Pigs were electrically stunned (320 V, 6 A for 10 s) and subsequently exsanguinated by jugular bleeding in accordance with current national slaughter regulations.

Carcass traits were evaluated according to ASPA guidelines [27]. Immediately after slaughter, hot carcass weight (HCW) was recorded after removal of the digestive, reproductive, and respiratory tracts, as well as perirenal fat. Carcass dressing percentage was calculated as the ratio between HCW and final body weight. Muscle pH was measured at 45 min (pH45) and 24 h (pH24) post mortem on the Longissimus thoracis et lumborum muscle, between the last thoracic and the first lumbar vertebra, using a portable penetration pH meter equipped with a temperature-compensated glass electrode (Hanna Instruments, San Benedetto del Tronto, Italy). The thickness of dorsal subcutaneous fat was measured on the carcass at the level of the last thoracic vertebra using a caliper, according to the method described by Chiofalo et al. [28].

### 2.7. Statistical Analysis

Due to differences in genotype, housing system, and trial duration, the two experimental trials were analyzed separately by SPSS statistical software (SPSS version 30.0, IBM Corp., Armonk, NY, USA).

#### 2.7.1. Purebred Apulo-Calabrese (AC)

Body weight (BW) and average daily gain (ADG) data were analyzed using Linear Mixed Models (LMM) to account for the hierarchical structure of the experiment and repeated measurements over time. Dietary treatments (CTR, SL, SLXI) were applied at pen level, with two pens per diet; therefore, Pen was specified as a random effect with a variance components covariance structure, and Pig was the observational unit for repeated measures. Fixed effects included Diet, Sex, Time (Timepoints: T0, T37, T71, T116, and T140 for BW; Periods: T0–T37, T37–T71, T71–T116, and T116–T140 for ADG) and their two- and three-way interactions. Repeated measurements within a pig were modeled using an unstructured covariance matrix for Time at the subject level. Degrees of freedom were estimated with the Satterthwaite method, and REML was used for parameter estimation. Estimated marginal means were compared with a Bonferroni adjustment.

Carcass traits and overall ADG, calculated from T0 to T140, were analyzed with analogous LMM including Diet and Sex as fixed effects and Pen as a random effect.

#### 2.7.2. Crossbred Apulo-Calabrese × Goland (AC × G)

In the crossbred trial, dietary treatments were applied at the pen level but only one pen per diet was available; thus, diet could not be treated as an inferential factor to avoid pseudoreplication. BW and ADG of crossbred pigs were analyzed using LMMs with Pig as the repeated subject and Time (Timepoints: T0, T18, T35, T56, T72, and T97 for BW; Periods: T0–T18, T18–T35, T56–T72, and T72–T97 for ADG), Sex and Sex × Time interaction as fixed effects. Repeated measurements within pig were modeled using an unstructured covariance matrix for Time at the subject level. Dietary groups are presented descriptively (means ± SD), without statistical testing of dietary effects.

Carcass traits and overall ADG, calculated from T0 to T197, were analyzed using LMM including Sex and Sex × Time as fixed effects, without random pen effect, as pen was not replicated. For both trials, all models were validated through residual diagnostics (histogram with normal curve, Q–Q plot, residuals vs. fitted values and residuals vs. time), confirming approximate normality and homoscedasticity.

Results, unless otherwise indicated, are presented as estimated marginal means ± standard error of the mean. Statistical significance was declared at *p* < 0.05.

## 3. Results

### 3.1. Growth Performance

In both feeding trials, the diets were completely consumed throughout the experimental period, with no feed refusals, indicating good palatability of all dietary treatments.

#### 3.1.1. Purebred AC Pigs

One purebred AC pig from the CTR group was excluded on T71 from the respective trial for reasons unrelated to the experiment, and all of its data were retained until this timepoint. BW and ADG are reported in Table 3.

**Figure 1:**
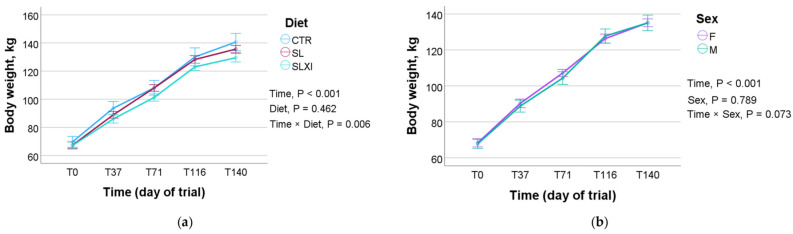
Body weight (mean ± SE) of purebred Apulo-Calabrese pigs (**a**) according to dietary treatment (CTR = control diet; SL = diet supplemented with 5% extruded linseed; SLXI = diet supplemented with 5% extruded linseed + 0.2% grape pomace powder) and (**b**) according to sex (female, male) across experimental time points.

Body weight increased progressively over time in all groups, from an initial mean value of 68.2 kg at T0 to 135.1 kg at T140. According to the Linear Mixed Model (Table 4), Time had a highly significant effect on BW (*p* < 0.001), whereas Diet, Sex, and their interactions did not significantly influence this parameter (Diet: *p* = 0.462; Sex: *p* = 0.789; Diet × Sex: *p* = 0.613). A significant Time × Diet interaction (*p* = 0.006) indicates that the temporal pattern of BW differed slightly among diets, although these differences did not translate into significant effects on overall body weight development (Figure 1a).

Sex did not significantly affect BW (*p* = 0.789), but a Time × Sex interaction approached significance (*p* = 0.073). Females and males exhibited similar growth trajectories, with both groups showing a steady increase in BW from T0 to T140 (Figure 1b).

These results indicate that neither linseed supplementation nor the combined inclusion of linseed and grape pomace powder impaired body weight development in purebred Apulo-Calabrese pigs.

Average daily gain (ADG) was also evaluated using an LMM including Diet, Sex, Time, and their interactions (Table 4). The model revealed a significant effect of Diet (*p* = 0.034), with SLXI pigs showing lower overall ADG compared with CTR animals (Table 3). Pairwise comparisons confirmed that SLXI differed significantly from CTR (*p* = 0.033), whereas SL did not differ from CTR (*p* = 0.953) nor from SLXI (*p* = 0.282). Sex did not significantly influence ADG (*p* = 0.577), although a Time × Sex interaction was detected (*p* = 0.047), indicating slightly different temporal patterns between females and males.

**Figure 2:**
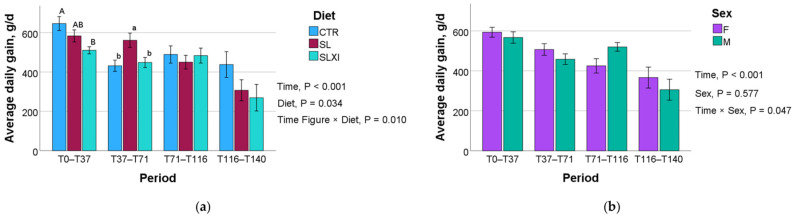
Average daily gain (mean ± SE) of purebred Apulo-Calabrese pigs (**a**) according to dietary treatment (CTR = control diet; SL = diet supplemented with 5% extruded linseed; SLXI = diet supplemented with 5% extruded linseed + 0.2% grape pomace powder) and (**b**) according to sex (female, male) across experimental periods. Different letters indicate significant differences among diets within each period (A, B = *p* < 0.01; a, b = *p* < 0.05; Bonferroni adjustment).

As expected in heavy pigs, ADG varied markedly across periods (*p* < 0.001). The highest growth rate occurred during the first phase (T0–T37; 580 g/day), followed by intermediate values in T37–T71 (483 g/day) and T71–T116 (478 g/day), and a pronounced reduction during the final finishing phase (T116–T140; 335 g/day). All the pairwise contrasts among periods were significant, confirming the physiological decline in growth rate as pigs approached slaughter weight. A significant Diet × Time interaction (*p* = 0.010) indicated that dietary effects on ADG were not constant across growth phases. Pairwise comparisons (Bonferroni-adjusted) revealed that average daily gain (ADG) differed significantly among diets during the first two periods (T0–T37 and T37–T71). Pigs fed the SLXI diet showed lower ADG than CTR in the initial period (*p* = 0.008), whereas SL-fed pigs exhibited higher ADG than both CTR and SLXI in the second period (*p* = 0.031 and *p* = 0.043, respectively). No significant differences were detected thereafter (*p* > 0.05) (Figure 2a).

As shown in Figure 2b, ADG was not significantly affected by Sex (*p* = 0.577), although a Time × Sex interaction was detected (*p* = 0.047). Estimated marginal means indicated that females tended to have higher ADG than males during the first two periods (T0–T37 and T37–T71), whereas males showed numerically greater values during T71–T116. However, pairwise comparisons adjusted by Bonferroni revealed no significant differences between sexes within any period (*p* > 0.05). These results suggest that growth dynamics differed slightly between the sexes over time, but without consistent statistical significance.

As previously mentioned, in all groups, the amount of feed consumed corresponded to the amount offered throughout the trial. However, because individual feed intake was not recorded, only mean reference values for feed intake and FCR are reported (Table 5).

#### 3.1.2. Crossbred AC × G Pigs

Because each dietary treatment was represented by a single pen, Diet could not be included as a fixed effect in the model, as this would violate the assumption of independent experimental units and lead to pseudoreplication. Therefore, BW and ADG values for the three dietary groups (CTR, SL, SLXI) are reported only descriptively, without statistical inference (Table 6).

**Figure 3:**
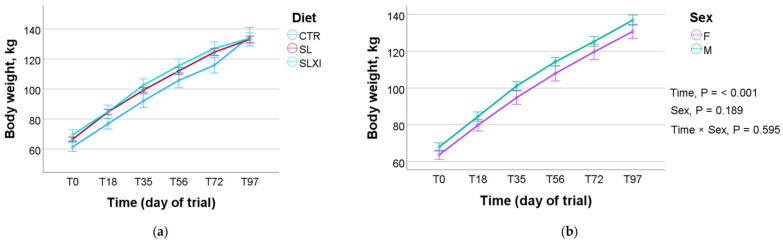
Body weight (mean ± SE) of crossbred Apulo-Calabrese × Goland pigs. (**a**) Descriptive trends by dietary treatment (CTR = control diet; SL = diet supplemented with 5% extruded linseed; SLXI = diet supplemented with 5% extruded linseed + 0.2% grape pomace powder) are shown for illustrative purposes only. (**b**) Body weight by sex (female, male) according to the Linear Mixed Model.

Body weight increased steadily over time in all crossbred pigs, rising from an overall mean of 65.8 kg at the beginning of the trial to 133.9 kg at slaughter. Time had a highly significant effect on BW (*p* < 0.001), confirming the expected growth pattern throughout the production cycle. In contrast, Sex and the Sex × Time interaction did not significantly influence BW trajectories (Figure 3).

**Figure 4:**
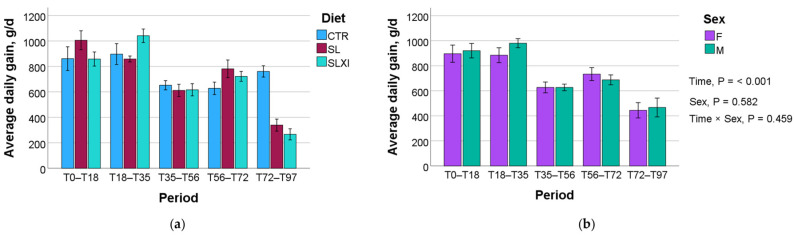
Average daily gain (mean ± SE) of crossbred Apulo-Calabrese × Goland pigs. (**a**) Descriptive values by dietary treatment (CTR = control diet; SL = diet supplemented with 5% extruded linseed; SLXI = diet supplemented with 5% extruded linseed + 0.2% grape pomace powder), shown for illustrative purposes only; (**b**) ADG by sex (female, male) according to the Linear Mixed Model.

ADG showed a similar pattern. The mixed model detected a significant effect of Period (*p* < 0.001), reflecting the physiological reduction in growth rate during the finishing phase. Neither Sex nor the Sex × Period interaction significantly affected ADG (Figure 4).

Table 7 reports the data on feed intake and feed conversion ratio observed in Apulo-Calabrese × Goland crossbred pigs. As in the case of the purebred trial, it should be noted that individual feed intake was not recorded; only group-level intake was measured. Therefore, these values should be regarded as indicative.

### 3.2. Carcass Traits

#### 3.2.1. Purebred AC Pigs

Carcass traits of purebred Apulo-Calabrese pigs are reported in Table 8. Dietary supplementation with extruded linseed, alone or combined with grape pomace powder, did not significantly affect any of the evaluated slaughter parameters.

Hot carcass weight (HCW) was comparable among groups (*p* = 0.335), indicating that the different dietary treatments did not influence carcass development at slaughter.

Similarly, carcass yield was not affected by diet (*p* = 0.650), with comparable dressing percentages observed among CTR, SL, and SLXI pigs.

Muscle pH measured at 45 min post mortem (pH45) did not differ among treatments (*p* = 0.979), indicating similar early post-mortem glycolytic evolution in all groups. Likewise, ultimate pH measured after 24 h of refrigerated storage (pH24) was not influenced by dietary supplementation (*p* = 0.574).

Backfat thickness was also unaffected by diet (*p* = 0.129). Finally, as shown in Table 8, neither Sex nor the Diet × Sex interaction significantly influenced any carcass trait in AC purebred pigs.

#### 3.2.2. Crossbred AC × G Pigs

Carcass traits of Apulo-Calabrese × Goland crossbred pigs for the three dietary groups are shown in Table 9. Because each dietary treatment corresponded to only one pen, Diet could not be included as a fixed factor in the statistical model. Consequently, the data are presented solely in a descriptive manner.

When evaluating the effect of Sex in the LMM (Table 10), a tendency was detected for HCW (*p* = 0.053), with males showing numerically higher values than females.

Carcass yield was significantly affected by Sex (*p* = 0.004), with male pigs showing higher dressing percentages than females.

Finally, regarding other characteristics of the carcass, no significant differences emerged between the two sexes.

## 4. Discussion

The present study evaluated the effects of dietary supplementation with extruded linseed, alone or combined with a low inclusion level of grape pomace powder rich in polyphenols, on growth performance and carcass traits in purebred Apulo-Calabrese pigs and Apulo-Calabrese × Goland crossbred pigs. Because the two trials differed in genetic background, housing system, age at the beginning of the experiment, trial duration, and timing of body weight measurements, they were analyzed and interpreted separately. Therefore, the observed differences between purebred and crossbred pigs should not be interpreted as direct genotype effects, but rather as responses observed within two distinct production contexts.

It should be emphasized that the dietary results obtained in crossbred pigs are exploratory, as the absence of replication at the pen level prevents formal statistical inference. Consequently, any apparent differences among dietary groups must be interpreted with caution. A major finding of the study was that the experimental diets were completely consumed in both trials, indicating good feed acceptance. This is relevant because grape pomace and other polyphenol-rich by-products may sometimes reduce palatability or nutrient utilization when included at high dietary levels, mainly because of their tannin content [13,14].

### 4.1. Purebred Apulo-Calabrese Pigs

In purebred pigs, growth performance followed the expected temporal pattern for heavy, slow-growing genotypes, with higher ADG during early phases and a progressive decline toward slaughter weight [28,29]. The mixed-model analysis indicated that dietary linseed did not impair growth, whereas the combined linseed + grape pomace diet (SLXI) produced a moderate reduction in overall ADG without affecting final BW.

Interestingly, this reduction was not accompanied by differences in final body weight or carcass characteristics, suggesting that its biological relevance was limited under the conditions of the present study. Rather than indicating an impairment of productive performance, these findings may reflect a slight modulation of growth dynamics across the fattening period.

This interpretation is further supported by the significant Diet × Time interaction observed for ADG. Pairwise comparisons indicated that the lower ADG of SLXI pigs was mainly confined to the initial phase of the trial, whereas pigs fed the SL diet exhibited the highest growth rate during the second growth period. Thereafter, no significant differences among dietary treatments were detected. These results suggest that the response to dietary supplementation was phase-dependent rather than constant throughout the production cycle. These findings are consistent with previous studies showing that linseed supplementation can be used in pig diets without compromising growth performance [28,29,30]. In local or slow-growing pig breeds, dietary linseed has been reported to improve the fatty acid profile of meat and fat without necessarily altering live weight gain or carcass development [31,32,33,34]. Similarly, studies on grape pomace supplementation in finishing pigs have reported variable effects depending on inclusion level, processing method, and physiological stage [35].

Although the combined supplementation slightly reduced overall ADG, neither body weight at slaughter nor carcass traits were negatively affected, indicating that the practical impact of this response was limited. This suggests that neither linseed nor grape pomace interfered with carcass development or tissue deposition in this local breed [36].

These findings are consistent with previous studies reporting that moderate inclusion levels of linseed do not compromise carcass traits in traditional or slow-growing pig genotypes [17,37], and that low-dose grape pomace supplementation may exert antioxidant benefits without inducing anti-nutritional effects [15,37]. The results on Sex and on the Time × Sex interaction suggest that the two sexes followed slightly different growth trajectories over time, while maintaining comparable overall productive performance.

### 4.2. Crossbred Apulo-Calabrese × Goland Pigs

In Apulo-Calabrese × Goland crossbred pigs, BW and ADG increased markedly during the trial and were significantly affected by time, reflecting the expected growth trajectory of slow-growing genotypes [30,38]. Sex did not significantly affect BW or ADG in either trial, and no relevant diet × sex interaction was observed for growth traits. This indicates that males and females responded similarly to the dietary treatments in terms of body weight development and average daily gain. Therefore, under the present experimental conditions, sex was not a major source of variability in growth performance.

Because dietary treatments were applied to single pens, diet could not be tested inferentially; therefore, all dietary results in crossbred pigs must be interpreted descriptively only. Numerical trends suggested phase-dependent differences among dietary groups, particularly during intermediate growth stages. However, these patterns did not persist until slaughter, and final BW was comparable across groups. The absence of inferential testing and the limited sample size prevent conclusions regarding dietary effects. Nonetheless, the stability of growth trajectories across treatments suggests that both linseed and grape pomace were well tolerated in this production context. However, because feed intake and FCR were derived from group-level data, mechanisms related to feed efficiency should be interpreted cautiously.

Carcass traits showed some descriptive variation among dietary groups, but inferential interpretation is not appropriate due to the lack of pen replication.

Sex significantly influenced carcass yield, with males showing higher dressing percentages than females. This result suggests a greater efficiency in converting live weight into carcass weight in male pigs under the conditions of the present study. Differences in dressing percentage between sexes have previously been reported in growing-finishing pigs and are generally associated with differences in body composition and the relative proportion of non-carcass components at slaughter rather than differences in growth performance itself [29,39].

A tendency towards higher hot carcass weight was also observed in males (*p* = 0.053), although the difference did not reach statistical significance. Considering that body weight at slaughter was comparable between sexes, this finding is consistent with the higher carcass yield observed in males and may reflect small differences in carcass composition rather than overall body development.

Conversely, muscle pH measured at 45 min and 24 h post mortem, as well as backfat thickness, were not significantly affected by sex.

### 4.3. Feed Intake and Feed Conversion Ratio

Feed intake was complete in both genetic groups, supporting the palatability of the experimental diets. This is an important practical result, particularly for the SLXI diet, because polyphenol-rich by-products may sometimes reduce voluntary intake. However, because animals were group-fed, feed intake and FCR should be interpreted as descriptive group-level indicators rather than individual-animal response variables.

Descriptively, FCR followed the expected pattern of reduced feed efficiency during the finishing period, which is consistent with the physiological decline in growth efficiency as pigs approach slaughter weight and maintenance requirements progressively increase relative to lean tissue deposition [40]. The available data does not support a strong negative effect of either linseed or linseed plus grape pomace on feed utilization. However, because pen-level replication was limited, particularly in the crossbred trial, FCR should not be overinterpreted as a definitive treatment effect.

## 5. Conclusions

Under the conditions of this study, dietary supplementation with 5% extruded flaxseed, alone or in combination with 0.2% grape pomace powder, was successfully incorporated into the diet of purebred Apulo-Calabrese pigs without negatively affecting final body weight or carcass characteristics. Although the combined supplementation resulted in a slight reduction in average daily weight gain, this response did not translate into significant differences in production performance at slaughter, indicating a limited biological impact under the experimental conditions used.

In the Apulo-Calabrese × Goland cross trial, growth performance and carcass characteristics followed the expected physiological trend throughout the fattening period. Because the dietary treatments were not replicated at the pen level, dietary observations should be considered descriptive and exploratory, and inferential conclusions about the effects of the diet cannot be drawn.

Overall, the presented results support the practical feasibility of incorporating extruded linseed and low-dose grape marc powder into feeding strategies for local pig production systems, while promoting the sustainable valorization of agro-industrial by-products within a circular economy framework. However, some limitations of this study should be acknowledged. Specifically, the lack of pen-level replication in the crossbred pig trial prevented a formal statistical evaluation of the effects of the diet, while feed intake and feed conversion ratio were recorded at the group level, limiting individual assessment of feed efficiency. These aspects should be considered when interpreting the results.

Future studies should therefore adopt fully replicated experimental designs, particularly in crossbred pigs, to confirm the effects of these dietary strategies on production performance and carcass characteristics. Further research should also investigate their influence on the fatty acid composition, oxidative stability, antioxidant status, technological properties, sensory quality, and shelf life of fresh pork and traditionally processed products, together with an assessment of their economic and environmental sustainability in local pig production systems.

## Figures and Tables

**Table 1:** Chemical composition (as fed), total polyphenol content, and antioxidant activity of the experimental diets and feed supplements.

Item	Growing CTR	Growing SL	Growing SLXI	Finishing CTR	Finishing SL	Finishing SLXI	Extruded Linseed	Grape Pomace Powder
Moisture, %	11.64	9.79	10.56	10.80	10.23	10.83	5.20	8.02
CP, %	15.16	15.19	15.33	14.18	14.25	14.29	19.08	11.2
EE, %	3.79	4.25	4.24	3.53	4.42	4.40	26.47	6.70
CF, %	5.08	5.40	5.40	5.85	6.17	6.15	7.52	24.4
Ash, %	4.95	5.15	5.15	4.91	5.04	5.00	5.19	4.90
ME, MJ/kg	13.4	13.4	13.4	13.2	13.2	13.2		
TPC, mg GAE/100 g	193	173	250	223	192	257	181	12,200
AA, %	1.08	0.58	1.36	0.74	0.77	0.97	0.63	33.12

CTR  = control diet; SL = diet supplemented with 5% extruded linseed; SLXI = diet supplemented with 5% extruded linseed and grape powdered pomace. CP = crude protein; EE = Ether extract; CF = Crude fiber; ME = metabolizable energy; TPC = total phenolic content expressed as mg Gallic Acid Equivalents (GAE)/100 g; AA = antioxidant activity expressed as percentage of DPPH inhibition.

**Table 2:** Fatty acid profile of the experimental diets and feed supplements.

Fatty Acid	Growing CTR	Growing SL	Growing SLXI	Finishing CTR	Finishing SL	Finishing SLXI	Extruded Linseed	Grape Pomace Powder
C14:0	0.00	0.00	0.00	0.00	0.00	0.00	0.00	6.56
C16:0	14.64	12.36	12.00	15.64	11.94	11.41	6.70	44.85
C16:1	0.25	0.15	0.19	0.22	0.28	0.31	0.00	0.65
C18:0	2.34	2.05	2.04	1.87	2.21	2.19	2.78	6.13
C18:1 n-9	24.20	21.88	22.01	26.17	21.88	22.05	13.90	34.71
C18:2 n-6	54.16	39.13	42.28	51.91	38.30	41.86	17.15	6.09
C18:3 n-3	3.23	23.29	20.08	2.68	23.84	20.53	59.03	0.45
C20:0	0.42	0.30	0.50	0.69	0.50	0.53	0.22	0.56
C20:1 n-9	0.54	0.47	0.54	0.58	0.63	0.73	0.22	0.00
C22:1 n-9	0.22	0.38	0.36	0.24	0.42	0.39	0.00	0.00
SFA	17.40	14.70	14.54	18.20	14.65	14.13	9.70	58.10
MUFA	24.99	22.50	22.74	26.97	22.79	23.09	14.12	35.36
PUFA	57.39	62.42	62.36	54.59	62.14	62.39	76.18	6.54

Fatty acids are expressed as a percentage of the total identified fatty acids. CTR = control diet; SL = diet supplemented with 5% extruded linseed; SLXI = diet supplemented with 5% extruded linseed and grape pomace powder; SFA = saturated fatty acids; MUFA = monounsaturated fatty acids; PUFA = polyunsaturated fatty acids.

**Table 3:** Body weight (kg) and Average daily gain (ADG, g/day) of purebred Apulo-Calabrese pigs fed experimental diets.

Item	Time Point	CTR (*n* = 11)	SL (*n* = 12)	SLXI (*n* = 12)
Body weight, kg	T0	69.7 ± 10.7	67.3 ± 10.8	67.1 ± 10.8
	T37	93.6 ± 10.9	88.9 ± 10.9	86.0 ± 10.9
	T71	108.8 ± 10.9	108.0 ± 10.9	101.3 ± 10.9
	T116	131.5 ± 11.2	128.2 ± 11.1	123.0 ± 11.1
	T140	141.7 ± 11.1	135.6 ± 11.1	129.5 ± 11.1
ADG, g/day	T0–T37	646 ± 215	584 ± 215	510 ± 215
	T37–T71	440 ± 216	561 ± 215	449 ± 215
	T71–T116	501 ± 216	450 ± 217	483 ± 217
	T116–T140	428 ± 223	307 ± 222	269 ± 222
	T0–T140	504 ± 214 ^a^	475 ± 214 ^ab^	427 ± 214 ^b^

CTR = control diet; SL = diet supplemented with 5% extruded linseed; SLXI = diet supplemented with 5% extruded linseed + 0.2% grape pomace powder. Values are estimated marginal means ± SE. Different superscript letters within a row indicate significant differences among dietary treatments (*p* < 0.05).

**Table 4:** Model *p*-values for Linear Mixed Model (LMM) of BW and ADG of Purebred Apulo-Calabrese pigs.

Item	Diet	Sex	Time	Diet × Sex	Time × Diet	Time × Sex	Time × Diet × Sex
BW	0.462	0.789	<0.001	0.613	0.006	0.073	0.650
ADG	0.034	0.577	<0.001	0.490	0.010	0.047	0.561

BW = body weight; ADG = average daily gain.

**Table 5:** Descriptive feed intake and feed conversion ratio (FCR) of purebred Apulo-Calabrese pigs by production phase for the experimental diets: CTR = control diet; SL = diet supplemented with 5% extruded linseed; SLXI = diet supplemented with 5% extruded linseed + 0.2% grape pomace powder.

	CTR (*n* = 11)		SL (*n* = 12)		SLXI (*n* = 12)	
Period	Feed Intake, kg/Head/d	FCR	Feed Intake, kg/Head/d	FCR	Feed Intake, kg/Head/d	FCR
T0–T37	2.40	3.85	2.40	4.11	2.40	4.70
T37–T71	2.70	6.20	2.70	4.81	2.70	6.02
T71–T116	3.00	6.19	3.00	6.67	3.00	6.21
T116–T140	3.20	7.36	3.20	10.43	3.20	11.89
Overall	2.80	5.59	2.80	5.75	2.80	6.29

Feed intake and FCR were calculated at the group level. FCR was calculated as feed intake divided by the corresponding ADG; overall FCR was calculated as total feed intake over the trial divided by BW gain from T0 to slaughter.

**Table 6:** Descriptive statistics of Body weight (BW, kg) and Average daily gain (ADG, g/day) of crossbred Apulo-Calabrese × Goland pigs fed experimental diets.

Item	Time Point	CTR (*n* = 10)	SL (*n* = 10)	SLXI (*n* = 10)
BW, kg	T0	61.4 ± 9.2	66.5 ± 4.6	69.5 ± 11.2
	T18	76.8 ± 11.1	84.7 ± 5.7	84.9 ± 13.9
	T35	92.1 ± 14.2	99.2 ± 5.9	102.6 ± 13.2
	T56	105.8 ± 15.6	112.1 ± 7.2	115.5 ± 14.2
	T72	115.8 ± 16.4	124.6 ± 7.5	127.1 ± 13.9
	T97	134.9 ± 19.6	133.1 ± 6.7	133.8 ± 11.6
ADG, g/day	T0–T18	861 ± 295	1006 ± 236	858 ± 175
	T18–T35	897 ± 259	859 ± 73	1041 ± 168
	T35–T56	652 ± 114	612 ± 152	617 ± 151
	T56–T72	628 ± 154	781 ± 219	722 ± 123
	T72–T97	761 ± 140	339 ± 149	267 ± 138
	T0–T97	760 ± 225	719 ± 286	701 ± 300

CTR = control diet; SL = diet supplemented with 5% extruded linseed; SLXI = diet supplemented with 5% extruded linseed + 0.2% grape pomace powder. Values are Mean ± Standard deviation.

**Table 7:** Descriptive feed intake and feed conversion ratio (FCR) of crossbred Apulo-Calabrese × Goland pigs by production phase for the experimental diets: CTR = control diet; SL = diet supplemented with 5% extruded linseed; SLXI = diet supplemented with 5% extruded linseed + 0.2% grape pomace powder.

	CTR (*n* = 10)		SL (*n* = 10)		SLXI (*n* = 10)	
Period	Feed Intake, kg/Head/d	FCR	Feed Intake, kg/Head/d	FCR	Feed Intake, kg/Head/d	FCR
T0–T18	2.26	2.62	2.26	2.25	2.26	2.63
T18–T35	2.40	2.68	2.40	2.79	2.40	2.31
T35–T56	2.40	3.68	2.40	3.92	2.40	3.89
T56–T72	2.80	4.46	2.80	3.58	2.80	3.88
T72–T97	2.80	3.68	2.80	8.26	2.80	10.50
Overall	2.54	3.35	2.54	3.71	2.54	3.84

Feed intake and FCR were calculated at group level. FCR was calculated as feed intake divided by the corresponding ADG; overall FCR was calculated as total feed intake over the trial divided by BW gain from T0 to slaughter.

**Table 8:** Carcass traits of purebred female (F) and male (M) Apulo-Calabrese pigs fed experimental diets.

Trait	CTR (*n* = 11)	SL (*n* = 12)	SLXI (*n* = 12)	Diet	F (*n* = 17)	M (*n* = 18)	Sex	Diet × Sex
				*p*			*p*	*p*
HCW, kg	110.8 ± 3.7	107.0 ± 3.5	103.1 ± 3.5	0.335	107.5 ± 3.0	106.4 ± 2.9	0.805	0.377
CY, %	78.74 ± 0.77	78.96 ± 0.73	79.67 ± 0.73	0.650	79.48 ± 0.62	78.76 ± 0.60	0.408	0.424
pH45	6.28 ± 0.07	6.26 ± 0.06	6.26 ± 0.06	0.979	6.25 ± 0.05	6.28 ± 0.05	0.651	0.452
pH24	6.03 ± 0.14	5.84 ± 0.13	5.87 ± 0.13	0.574	5.86 ± 0.11	5.97 ± 0.11	0.476	0.374
BFT, mm	43.3 ± 2.3	36.6 ± 2.2	40.0 ± 2.2	0.129	42.0 ± 1.9	37.9 ± 1.8	0.126	0.541

CTR = control diet; SL = diet supplemented with 5% extruded linseed; SLXI = diet supplemented with 5% extruded linseed + 0.2% grape pomace powder. HCW = hot carcass weight; CY = carcass yield; BFT = backfat thickness. Values are estimated marginal means ± SE.

**Table 9:** Descriptive statistics of carcass traits in crossbred Apulo-Calabrese × Goland pigs fed experimental diets.

Trait	CTR (*n* = 10)	SL (*n* = 10)	SLXI (*n* = 10)
Hot carcass weight, kg	103.8 ± 16.5	105.9 ± 6.2	109.2 ± 10.7
Carcass yield, %	79.55 ± 2.28	80.79 ± 1.75	82.66 ± 2.90
pH45	6.30 ± 0.20	6.51 ± 0.20	6.16 ± 0.20
pH24	5.55 ± 0.40	5.20 ± 0.45	5.45 ± 0.48
Backfat thickness, mm	32.3 ± 6.7	32.9 ± 3.5	31.1 ± 6.3

CTR = control diet; SL = diet supplemented with 5% extruded linseed; SLXI = diet supplemented with 5% extruded linseed + 0.2% grape pomace powder. Values are Mean ± Standard deviation.

**Table 10:** Carcass traits of crossbred female (F) and male (M) Apulo-Calabrese × Goland pigs.

Trait	F (*n* = 15)	M (*n* = 15)	Sex
			*p*
Hot carcass weight, kg	102.2 ± 2.9	110.4 ± 2.9	0.053
Carcass yield, %	79.67 ± 0.59 ^B^	82.33 ± 0.59 ^A^	0.004
pH45	6.27 ± 0.06	6.38 ± 0.06	0.232
pH24	5.44 ± 0.12	5.37 ± 0.12	0.674
Backfat thickness, mm	30.9 ± 1.5	33.2 ± 1.4	0.262

Values are estimated marginal means ± Standard error. Different superscript letters within a row indicate significant differences between the two sexes (*p* < 0.01).

## Data Availability

The raw data supporting the conclusions of this article will be made available by the authors on request.

## Abbreviations

The following abbreviations are used in this manuscript:

AC	Apulo-Calabrese
AC × G	Apulo-Calabrese × Goland
CTR	Control diet
SL	Diet supplemented with 5% extruded linseed
SLXI	Diet supplemented with 5% extruded linseed and 0.2% grape pomace powder
BW	Body weight
ADG	Average daily gain
FCR	Feed conversion ratio
HCW	Hot carcass weight
CY	Carcass yield
SE	Standard error of the mean
pH45	pH measured 45 min post mortem
pH24	pH measured 24 h post mortem
